# Discriminating Origin Tissues of Tumor Cell Lines by Methylation Signatures and Dys-Methylated Rules

**DOI:** 10.3389/fbioe.2020.00507

**Published:** 2020-05-26

**Authors:** Shiqi Zhang, Tao Zeng, Bin Hu, Yu-Hang Zhang, Kaiyan Feng, Lei Chen, Zhibin Niu, Jianhao Li, Tao Huang, Yu-Dong Cai

**Affiliations:** ^1^School of Life Sciences, Shanghai University, Shanghai, China; ^2^Department of Biostatistics, University of Copenhagen, Copenhagen, Denmark; ^3^Shanghai Research Center for Brain Science and Brain-Inspired Intelligence, Shanghai, China; ^4^State Key Laboratory of Livestock and Poultry Breeding, Guangdong Public Laboratory of Animal Breeding and Nutrition, Guangdong Key Laboratory of Animal Breeding and Nutrition, Institute of Animal Science, Guangdong Academy of Agricultural Sciences, Guangzhou, China; ^5^Shanghai Institute of Nutrition and Health, Shanghai Institutes for Biological Sciences, Chinese Academy of Sciences, Shanghai, China; ^6^Department of Computer Science, Guangdong AIB Polytechnic, Guangzhou, China; ^7^College of Information Engineering, Shanghai Maritime University, Shanghai, China; ^8^College of Intelligence and Computing, Tianjin University, Tianjin, China

**Keywords:** methylation signature, dys-methylated pattern, cell line, rule, classification

## Abstract

DNA methylation is an essential epigenetic modification for multiple biological processes. DNA methylation in mammals acts as an epigenetic mark of transcriptional repression. Aberrant levels of DNA methylation can be observed in various types of tumor cells. Thus, DNA methylation has attracted considerable attention among researchers to provide new and feasible tumor therapies. Conventional studies considered single-gene methylation or specific loci as biomarkers for tumorigenesis. However, genome-scale methylated modification has not been completely investigated. Thus, we proposed and compared two novel computational approaches based on multiple machine learning algorithms for the qualitative and quantitative analyses of methylation-associated genes and their dys-methylated patterns. This study contributes to the identification of novel effective genes and the establishment of optimal quantitative rules for aberrant methylation distinguishing tumor cells with different origin tissues.

## Introduction

DNA methylation is an essential epigenetic modification for multiple biological processes (Gao et al., 2017). It is characterized by the formation of 5-methylcytosine in the CpG site with the control of DNA methyltransferases (Moore et al., 2013). Recent studies have discovered that non-CpG methylation functions as an expression regulator in mammals (Guo et al., 2014; Zhang et al., 2017). However, the primary role of this process in mammals remains elusive. Since DNA methylation was considered a regulator in gene expression in the 1970's (Holliday and Pugh, 1975), numerous studies have investigated methylation-associated mechanisms, and functions. Ample solid evidence suggests that DNA methylation is involved in essential developmental events, such as X-chromosome inactivation and genomic imprinting. Current knowledge is that DNA methylation in mammals acts as an epigenetic mark of transcriptional repression.

During pathologic progression, tumors are deemed to be a genetic, and epigenetic disease. Classic genetic and epigenetic alterations co-determine tumor initiation and progression (Zhou et al., 2016). Aberrant levels of DNA methylation can be observed in various types of tumor cells. With the increasing recognition of tumorigenesis, altered DNA methylation has been described as a basic “cancer driver” event (Campan et al., 2011) that can be divided into two types, namely, hypomethylation and hypermethylation. In general, the over-activation of proto-oncogenes caused by DNA hypomethylation is a major dysfunctional process during tumorigenesis (Renaud et al., 2015, 2016; Good et al., 2018). Meanwhile, abnormal hypermethylation in CpG islands of tumor suppressor gene promoter (e.g., PTEN and p16) could lead to gene silencing and tumor initiation (Marzese et al., 2014; Cui et al., 2015; De La Rosa et al., 2017). The methylation abnormally and indirectly induces tumorigenesis in other DNA regions, such as repetitive sequences (Hur et al., 2014; Burns, 2017; Chen et al., 2017c). Hence, studies on DNA methylation are warranted to provide new and feasible tumor therapies.

Divergent methylation patterns are intensely associated with cell differentiation (Farlik et al., 2016). Even in a single cell line, methylation patterns may be dynamic among different stages (Kaaij et al., 2013; Petell et al., 2016), and this situation is common for tumor cells. In accordance with the initial original organs and tissues, tumors can be divided into different subtypes with different genome-wide methylation patterns. Therefore, a part of particular methylation patterns should be recognized as epigenetic marks for specific tumor sites (Sahm et al., 2017). For example, mucin is a macromolecular glycoprotein secreted mainly by goblet cells, which act as a protective barrier (Pelaseyed et al., 2014), and hypomethylation of mucin gene MUC5AC is considered a feature in colorectal cancers (Renaud et al., 2015, 2016). Another research also reveals that BRCA1, an essential tumor-suppressor gene, is highly associated with breast and ovarian cancer when the promoter undergoes hypermethylation (Evans et al., 2018). Hence, DNA methylation is supposed to emerge as a tumor-specific marker with large potentiality.

Most conventional studies considered single-gene methylation or specific loci as biomarkers for tumorigenesis. However, the entire genome-scale methylated modification has not been fully revealed. Tumor is a typical type of disease with high heterogeneity and individual difference. Thus, the combination of multiple sites with methylation patterns can highly increase the accuracy and sensitivity of markers. Hence, in this study, we proposed and compared two novel computational approaches involving multiple algorithms, namely, Monte Carlo feature selection (MCFS; Draminski et al., 2008), minimum redundancy maximum relevance (mRMR; Peng et al., 2005), and repeated incremental pruning to produce error reduction (RIPPER; Cohen, 1995), for the qualitative and quantitative analyses of methylation-associated genes and their dys-methylated patterns. This study contributes to the identification of novel effective genes and the establishment of optimal quantitative rules for methylation distinguishing tumor cells with different origin tissues.

## Materials and Methods

### Dataset

We downloaded the methylation profiles of 1,022 cell lines from Gene Expression Omnibus under accession number GSE68379 (Iorio et al., 2016). In each cell line, the methylation levels of 485,512 probes were measured. We applied the KNN method to impute the missing values. The *R* function impute.knn from package impute (https://bioconductor.org/packages/impute/) was used, and *K* was set to 10. Of note, there were actually very few missing values in this dataset, where the highest missing value percentage of the samples was about 0.1%. Therefore, we used the default parameter of K (10) and did not try other values. The 1,022 cell lines were from 13 tissues, and the sample sizes of 13 tissues are listed in Table 1. We determined whether the cell lines from different tissues differ in methylation level.

**Table 1:** Sample sizes of 13 tissues.

**Index**	**Primary site**	**Sample size**
1	Aerodigestive Tract	80
2	Blood	177
3	Bone	38
4	Breast	52
5	Digestive system	105
6	Kidney	33
7	Lung	198
8	Nervous system	96
9	Pancreas	31
10	Skin	59
11	Soft tissue	21
12	Thyroid	17
13	Urogenital system	115

### Feature Selection

We proposed two novel feature selection schemes for detecting specific signatures to distinguish methylation-related genes in tumor cells. We use mRMR (Peng et al., 2005) and MCFS (Draminski et al., 2008) to evaluate each feature, select the candidate features, and then use the support vector machine (SVM; Cortes and Vapnik, 1995) and other alternative algorithms to train the subsets of features in the incremental feature selection (IFS; Liu and Setiono, 1998) to identify specific signatures for screening tumor cells.

#### Selection of Important Features

Each cell line is represented by more than 480,000 methylation features. Clearly, it is impossible that all of them are essential for classifying cell lines into correct tissues. Thus, we first adopt mutual information (MI) to select essential features. The mutual information (MI) between *x* and *y* is defined as follows:

(1) $$\begin{array}{r} I\left(x,y\right)=\iint p\left(x,y\right)\log\frac{p\left(x,y\right)}{p\left(x\right)p\left(y\right)}dxdy, \end{array}$$

where *p*(*x*) represents marginal probabilistic density of *x* and *p*(*x, y*) indicates joint probabilistic density of *x* and *y*. For each feature, the MI value to class labels is calculated. It is widely accepted that features with high MI values are highly related to class labels, thereby giving key contributions for classification. Thus, we can select important features by setting a threshold for MI value. Features with MI values higher than the threshold are selected for further evaluation. They will be assessed by the following two feature selection methods.

#### Minimum Redundancy and Maximum Relevance

Remaining features are analyzed by mRMR (Peng et al., 2005). As a feature filtering method, mRMR requires two optimal targets on the highest relevance among selected feature subsets, namely, the maximum relevance between feature sets and labels and the minimum redundancy between features themselves (Peng et al., 2005). Such evaluations are all based on MI values. The output of mRMR contains a feature list, which sorts features according to maximum relevance and minimum redundancy. The list is generated by selecting a feature with maximum relevance to labels and minimum redundancy to already-selected features one by one and adding it to the current feature list.

#### Monte Carlo Feature Selection

Remaining features are also evaluated by MCFS (Draminski et al., 2008). This method has been applied as a classical feature selection method for dealing with many biological problems. MCFS is a random sampling-based feature selection method. In specific, MCFS trains multiple decision trees in a bootstrap sample set and a subset of randomly selected features (e.g., *m* features from the original *M* features, and *m* < < *M*). For a specific feature subset, samples with this subset of features can compose *p* bootstrap training sets. Thus, *p* decision trees can be obtained through training and evaluation. Assuming that this process is repeated *t* times, we can finally obtain *p* × *t* decision trees.

Relative importance (RI) is a score used to define how features are performed in each constructed classifier from the *p* × *t* decision trees. The RI score for a feature *g* is calculated as follows:

(2) $$\begin{array}{r} RI_{g}=\overset{pt}{\underset{\tau =1}{\sum }}{\left(wAcc\right)}^{u}IG\left(n_{g}\left(\tau \right)\right)\left(\frac{no.in\;n_{g}\left(\tau \right)}{no.in\;\tau }\right)v, \end{array}$$

where wAcc is the weighted accuracy calculated by the mean sensitivity of all decision classes, *n*_*g*_(τ) is a node involving feature g in decision tree τ, *IG*(*n*_*g*_(τ)) is the information gain of *n*_*g*_(τ), *no*.*in τ* is the number of samples in decision tree τ, and *no*.*in n*_*g*_(τ) is the number of training samples in node *n*_*g*_(τ). In addition, *u* and *v* are two different weighting factors for adjusting different optimal contributions. After features has been assigned RI scores, a feature list can be generated by the decreasing order of their RI scores.

In this study, we used the MCFS program retrieved from http://www.ipipan.eu/staff/m.draminski/mcfs.html. Default parameters were used to execute such program, where *p* = 2000, *t* = 5, and *u* = *v* = 1.

#### Incremental Feature Selection

In the descending ordered feature list generated by MCFS or mRMR, we perform IFS to filter out a set of optimal features for accurately distinguishing different sample groups/classes (Liu and Setiono, 1998). We construct a series of feature subsets with an interval of 10 from the ranked feature list *F* by MCFS or mRMR. We generate *m* feature subsets $F_{1}^{1},F_{2}^{1},\ldots ,F_{m}^{1}$, where the *i*-th feature subset contains the top 10 × *i* features $F_{i}^{1}=\left[f_{1},f_{2},\ldots ,f_{i\times 10}\right]$in *F*. All feature subsets are tested by building and evaluating the SVM classifier (or other alternative methods such as rule-based approaches) using 10-fold cross-validation. The feature subset with the best performance is called the optimal feature subset.

### Supervised Classifier

The supervised classifiers for IFS include “black-box” classifier SVM, interpretable rule learning classifier RIPPER (Cohen, 1995), and PART algorithm (Frank and Witten, 1998).

#### Support Vector Machine

SVM is a supervised learning algorithm based on statistical learning theory (Cortes and Vapnik, 1995; Chen et al., 2017b, 2018a; Che et al., 2020; Zhou et al., 2020a,b). It uses kernel techniques (such as Gaussian kernels) to map the original data from a low-dimensional non-linear space to a high-dimensional linear space and then fits the hyperplane in the high-dimensional space with the largest margin between the two classes of samples by using a linear function. We use the sequential minimal optimization (SMO) algorithm in software Weka for SVM classifier training with default parameters. The kernel was a polynomial function, the regularization parameter *C* was one.

#### Rule Learning Classifier RIPPER

We also use RIPPER (Cohen, 1995), a learner proposed by William that can generate classification rules to classify samples from different tumor cells. RIPPER can learn interpretable classifications for predicting new data in accordance with IF-ELSE rules. RIPPER learns all rules for each sample class. After learning rules for one class, RIPPER moves to learn the rules for the next class. RIPPER starts from the minority sample class and then to the second minority sample class until the dominant class. The “JRip” tool, implementing RIPPER algorithm, in Weka is used. Default parameters are adopted, where the parameter to determine the amount of data used for pruning is set to three.

#### Rule Learning Classifier PART

Different from the RIPPER algorithm that builds a full decision tree, the PART algorithm (Frank and Witten, 1998) learns rules by repeatedly generating partial decision trees. It uses a separate-and-conquer strategy to build a rule, removes the instance covered by this rule, and continues to generate rules recursively until all instances are covered. Compared with RIPPER, PART is simpler and does not need any global optimization. To quickly implement PART algorithm, we directly use the tool “PART” in Weka.

### SMOTE

As indicated in Table 1, the analyzed dataset consists of different numbers of cell lines from different tissues; thus, it is an imbalanced data. Therefore, we use the synthetic minority over-sampling technique (SMOTE) to obtain approximate balanced data ahead of classifier construction (Chawla et al., 2002). SMOTE produces new samples for the minor class iteratively until the size of the minor class can be equal to that of the major class. The tool “SMOTE” in Weka is used to produce new samples for each minor class (tissue); thus, the numbers of cell lines for all tissues are equal finally, that is, the number of samples in each class (tissue) is 198. The main parameter that determines the number of nearest neighbors in the same class for a selected sample is set to three.

### Performance Measurement

As a balanced measurement, the Matthew's correlation coefficient (MCC; Matthews, 1975; Gorodkin, 2004) is used to evaluate and compare the classifier performance. Originally, MCC is designed for binary classification and has wide applications (Chen et al., 2017a,b; Zhao et al., 2018, 2019; Cui and Chen, 2019; Li et al., 2019), as proposed by Matthews in 1975 (Matthews, 1975). We adopte the multi-class version of MCC proposed by Gorodkin (Gorodkin, 2004) because our analyzed dataset contains more than two classes (i.e., tissues), and such MCC is calculated as follows:

(3) $$\begin{array}{r} MCC=\frac{\mathrm{cov}\left(X,Y\right)}{\sqrt{\mathrm{cov}\left(X,X\right)\mathrm{cov}\left(Y,Y\right)}}, \end{array}$$

where cov(·, ·) stands for the covariance of two matrices, *X* is a 0-1 matrix indicating the predicted class of each sample, and *Y* is a 0–1 matrix representing the actual classes of all samples. Such multi-class version of MCC has been widely used in the performance evaluation of multi-class classifiers (Salari et al., 2014; Schmuker et al., 2014; Zhang et al., 2019); thus, the multi-class version of MCC is still called MCC for convenience. In addition, we also report the accuracy of each class and over accuracy (ACC) for reference.

## Results

**Figure 1:**
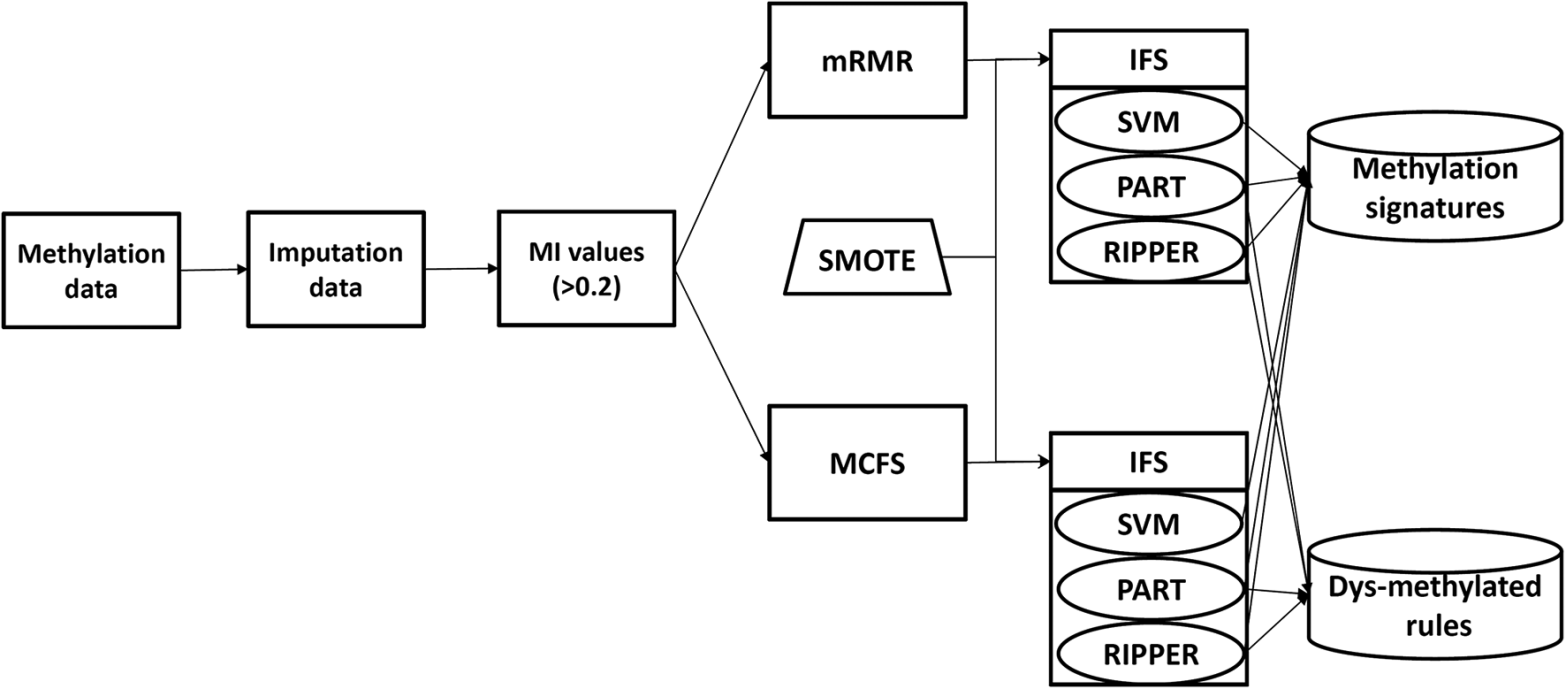
Analysis framework.

In this study, we analyze the methylation data of cell lines in 13 tissues. The entire procedures are shown in Figure 1. Of the 485,512 methylation features, we first calculate their MI values to class labels. By setting the threshold 0.2 to MI value, 20,451 features remain, which are provided in Table S1. Then, these features are analyzed by mRMR and MCFS methods, respectively, producing two feature lists, which are available in Tables S1, S2, respectively. Then, on the basis of each feature list, we use IFS combined with a particular classifier to determine the optimal feature set and related classification models or rules.

### Tumor Cell Classification Based on Ranked Features by mRMR

**Figure 2:**
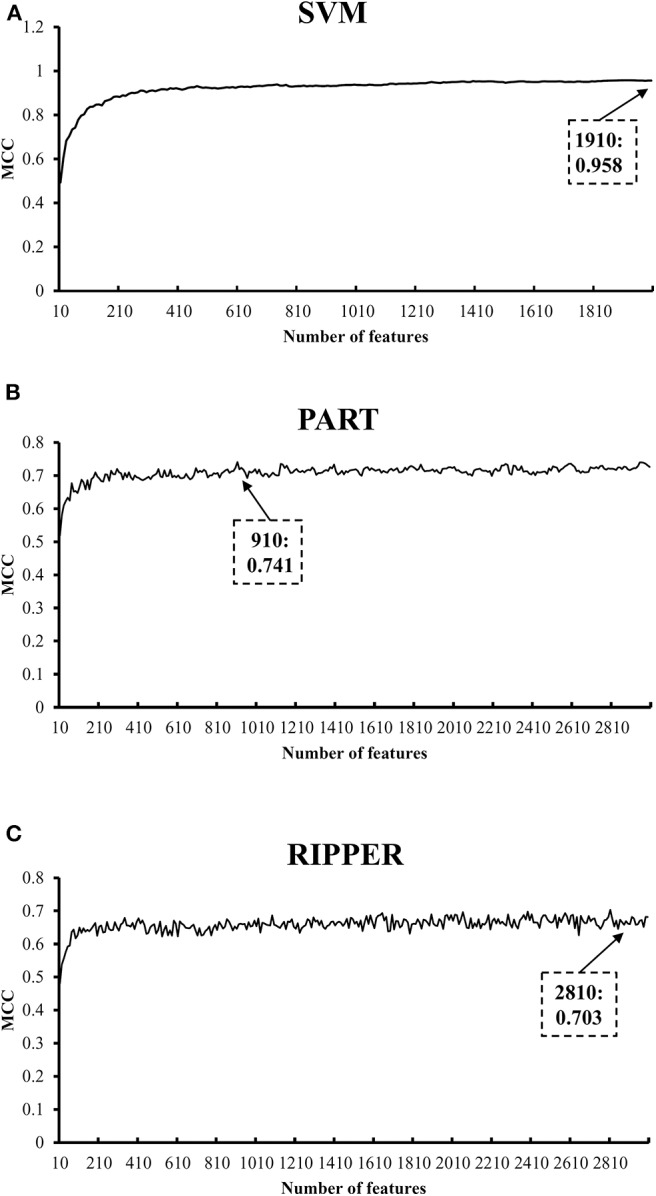
IFS curves with SVM, PART, and RIPPER based on mRMR-ranked features. **(A)** IFS with SVM. When top 1910 features are used, SVM gives the best MCC of 0.958. **(B)** IFS with PART. When top 910 features are used, PART gives the best MCC of 0.741. **(C)** IFS with RIPPER. When top 2810 features are used, RIPPER gives the best MCC of 0.703.

**Figure 3:**
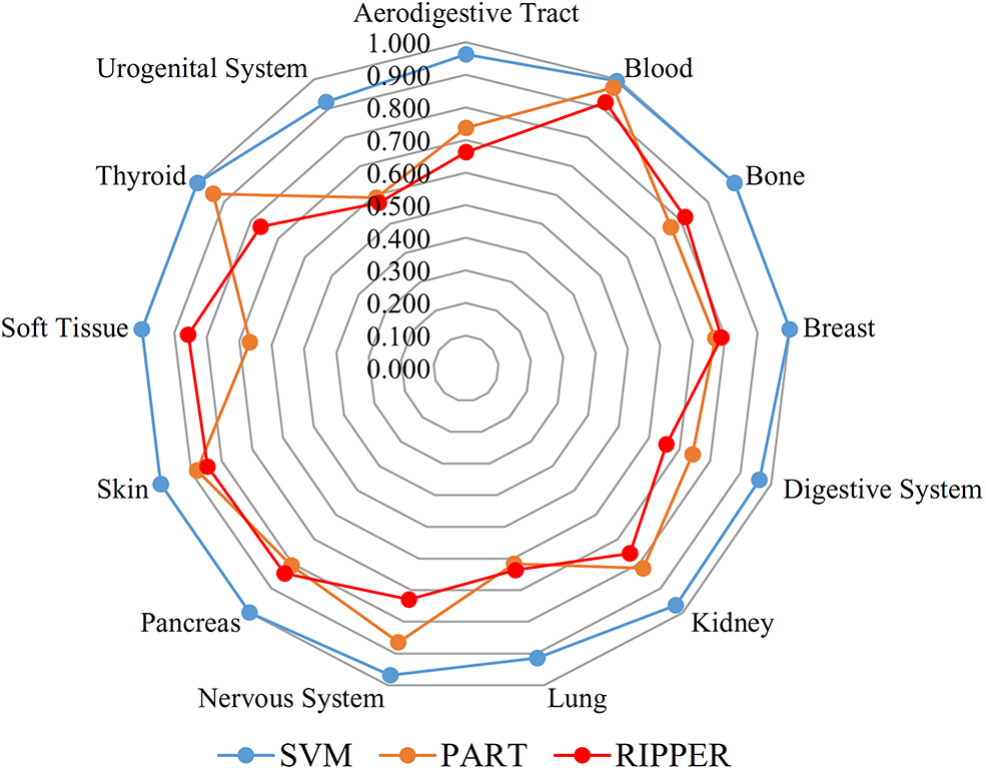
Radar chart to show the performance of the best SVM, PART and RIPPER classifiers on 13 tissues based on the feature list yielded by mRMR.

We initially generate a series of feature subsets from the ranked feature list by mRMR and then run the IFS with SVM, RIPPER, and PART to capture optimal features for classifying different tumor cell samples. The performance these classifiers with different numbers of features is listed in Table S3. For an easy observation, an IFS curve is plotted for each classifier with the number of features as X-axis and MCC as the Y-axis, as shown in Figure 2. The highest MCC value generated by the SVM is 0.958 when using top-ranked 1,910 features, the optimal MCC value generated by the PART is 0.741 when using top-ranked 910 features, and the best MCC obtained by RIPPER is 0.703 when using top-ranked 2,810 features. The ACCs corresponding to above MCCs are 0.963, 0.768, and 0.735, respectively. Above results are listed in Table 2. Furthermore, we also count the accuracy of each tissue yielded by above three classifiers, which are illustrated in Figure 3. All accuracies yielded by SVM are over 0.900, whereas only two and one tissues receive the accuracies over 0.900 for PART and RIPPER, respectively. All these results show that the “black-box” classifier SVM performs better than rule-based classifiers. However, rule-based classifiers can learn readable rules for making an interpretable prediction. The PART algorithm generates 72 classification rules, as shown in Table S4, and RIPPER learns 47 classification rules, as shown in Table S5.

**Table 2:** Performance of IFS with SVM, PART, and RIPPER based on mRMR-ranked features for classifying tumor cells from different tissues.

**Classifier**	**Number of optimal features**	**ACC**	**MCC**
SVM	1,910	0.963	0.958
PART	910	0.768	0.741
RIPPER	2,810	0.735	0.703

### Tumor Cell Classification Based on Ranked Features by MCFS

**Figure 4:**
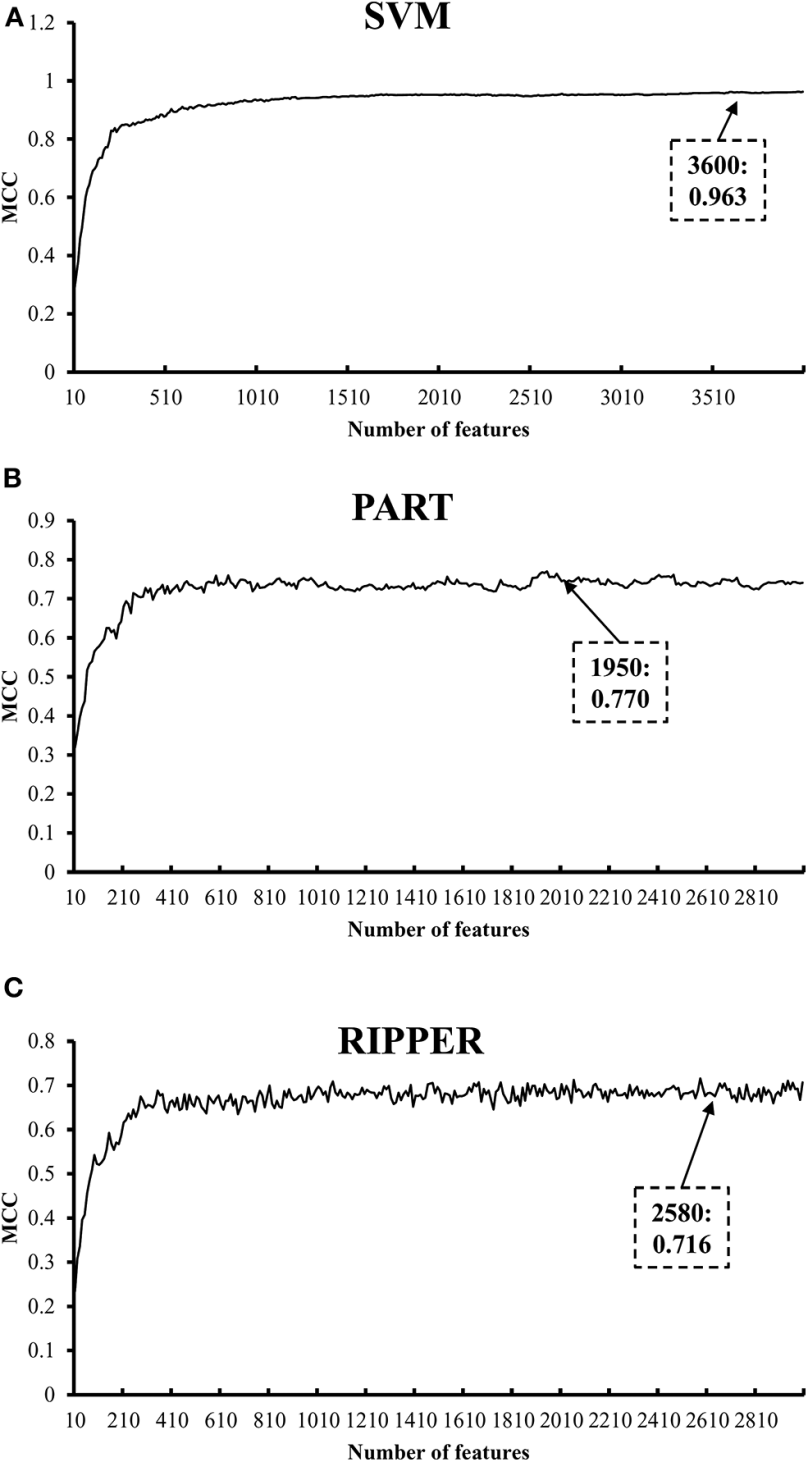
IFS curves with SVM, PART, and RIPPER based on MCFS-ranked features. **(A)** IFS with SVM. When top 3600 features are used, SVM gives the best MCC of 0.963. **(B)** IFS with PART. When top 1950 features are used, PART gives the best MCC of 0.770. **(C)** IFS with RIPPER. When top 2580 features are used, RIPPER gives the best MCC of 0.716.

**Figure 5:**
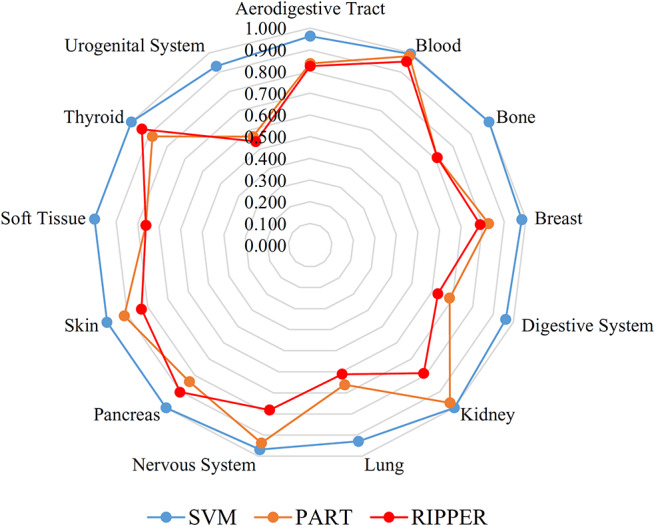
Radar chart to show the performance of the best SVM, PART, and RIPPER classifiers on 13 tissues based on the feature list yielded by MCFS.

We also carry out a similar analysis pipeline on the ranked features from MCFS. The performance of three classifiers on different numbers of features is listed in Table S6. Likewise, an IFS curve is plotted for each classifier, as shown in Figure 4. The best MCCs generated by SVM, PART, and RIPPER are 0.963, 0.770, and 0.716 when using top-ranked 3,600, 1,950, and 2,580 features, respectively, as listed in Table 3. The corresponding ACCs are 0.967, 0.795, and 0.746, respectively (see Table 3). Furthermore, the performance on 13 tissues of these three classifiers is shown in Figure 5. All accuracies generated by SVM are higher than 0.900, whereas for PART and RIPPER, there are only four and three accuracies over 0.900, respectively. SVM also outperforms rule learning classifiers PART and RIPPER. However, one advantage of PART and RIPPER is that they can learn interpretable rules for human understanding. PART learns 80 classification rules (Table S7), and RIPPER learns 48 classification rules (Table S8).

**Table 3:** Performance of IFS with SVM, RART, and RIPPER based on MCFS-ranked features for classifying tumor cells from different tissues.

**Classifier**	**Number of optimal features**	**ACC**	**MCC**
SVM	3,600	0.967	0.963
PART	1,950	0.795	0.770
RIPPER	2,580	0.746	0.716

## Discussion

Established by World Health Organization (WHO), the classification scheme of tumor has been amended several times over the past decades. Scholars attempt to analyze the major characteristic of each type of tumor to provide solid guidance for clinical diagnosis and to avoid misclassification with mimic entities. For instance, as the most common digestive tract malignancies, misdiagnosis of metastatic colorectal cancer is highly responsible for the primary resistance of immune checkpoint inhibitors, displaying microsatellite instability, or defective mismatch repair (Cohen et al., 2019). Furthermore, the diagnosis of tumor with the good deal of insight of DNA methylation should improve the preciseness compared with traditional methods (Sahm et al., 2017). In accordance with our approach and analysis, we detected various methylation patterns of genes and association rules in different cell lines that can be used as the candidate signatures to distinguish 13 tumor subgroups corresponding to particular origin tissues. All predicted candidate signatures have reported that the aberrant methylations occurred and attributed to tumor initiation and progression. A summary and discussion on these signatures are presented in the following section.

### Candidate Methylation Signatures Discriminating Origin Tissues of Tumor Cells

The first list of genes has been obtained by the MCFS and SVM algorithms. In accordance with the related results, **MIR142** was predicted as one of the most potential genes for tumor classification. In general, the dysfunctions of **MIR142** attribute to tumorigenesis and angiogenesis. **MIR142** specifically expresses and plays a critical role in various hematopoietic cell lines (Rivkin et al., 2017). Hypermethylation-induced silencing of **MIR142** promotes the progression of hepatocellular carcinoma via failing to suppress TGF-β expression (Yu et al., 2017). Similarly, the downregulation of **MIR142** induced by promoter hypermethylation participates in thyroid follicular tumor initiation (Colamaio et al., 2015). Recent relevant studies have also confirmed that DNA methylation in **MIR142** promoter can be recognized as a novel biomarker for T cell lymphoma (Sandoval et al., 2015). **BZRAP1** encodes an associated protein of translocator protein, which regulates the flow of cholesterol into mitochondria. Translocator protein presents different expression patterns in different types of tumor (Bhoola et al., 2018). Although few studies directly concentrated on the function of **BZRAP1** methylation, **BZRAP1** may be a potential marker for tumor classification considering the relationship between **BZRAP1** and translocator proteins. Another gene, **IFFO1**, is widely methylated in ovarian tumor. Compared with normal blood samples, significant hypo-methylation on **IFFO1** promoter is a potentially high-sensitive biomarker for ovarian tumor diagnosis. In addition, hyper-methylation of IFFO1 represses its expression in non-small-cell lung cancer (Feng et al., 2017).

Then, we applied another computational algorithm combining mRMR and SVM to predict differentially methylated gene candidates. The predictable ability of mRMR has been validated with high efficacy and accuracy. Recently, the mRMR algorithm has been applied to identify deriver genes of clear cell renal cell carcinoma (Li et al., 2018). We actually obtained a large group of tumor-associated methylated genes through the mRMR algorithm. Similar to the above MCFS method, **BZRAP1** and **IFFO1** also appeared in the feature list. Numerous studies have revealed the contribution of methylation on tumorigenesis, implying the accuracy and efficiency of our two analysis pipelines. On the basis of our results, **MARVELD2** was predicted to show methylation diversity in tumor cells. **MARVELD2** encodes an essential tight junction-associated member protein named “tricellulin.” In general, this protein expresses in tricellular junctions and contributes to the stability of epithelial cell layers. Hence, abnormal **MARVELD2** expression always associates with various types of carcinoma pathogenesis. Early in 2011, the expression of **MARVELD2** is evidently decreased in every stage of squamous cell carcinoma (Kondoh et al., 2011). Recent studies have further revealed that **MARVELD2** is frequently overexpressed in hepatocellular carcinoma cells but downregulated in pancreatic carcinomas cells (Kojima and Sawada, 2012; Korompay et al., 2012; Somoracz et al., 2014). In consideration of the relationship between gene expression and methylation, this evidence could suggest the methylation diversity of **MARVELD2** in different tumor types. **LDOC1** is an important tumor-suppressor gene that mainly contributes to the regulation of transcriptional response mediated by the nuclear factor kappa B (Griesinger et al., 2017). Hyper-methylation causes **LDOC1** silencing in multiple tumor types, such as cervical cancer (Buchholtz et al., 2013), lung cancer (Lee et al., 2019), and oral squamous cell carcinoma (Lee et al., 2013), implying the accuracy and efficacy of our prediction. **MGAT1**, a member of the glycosyltransferase family, acts as a Medial–Golgi enzyme that mediates the synthesis of complex N-glycans. A previous report confirmed that **MGAT1** contributes to tumor migration and invasion (Beheshti Zavareh et al., 2012). As an important obesity-associated gene (Johansson et al., 2010), differential methylation of **MGAT1** is associated with obesity risk (Voisin et al., 2015). Considering the strict relationship between obesity and the digestive system, **MGAT1** might act as a candidate methylated marker for the digestive system. Moreover, **MGAT1** is hyper-methylated in head and neck squamous cell carcinomas (Hwang et al., 2013). Another splicing regulator gene, **ESRP2**, was also predicted to present methylation diversity in tumor cells. In general, such gene is mainly expressed in various types of epithelial cells. For its particular methylation status, **ESRP2** is overexpressed as induced by gene hypo-methylation in ovarian cancer and breast cancer (Heilmann et al., 2017; Jeong et al., 2017). Therefore, **ESRP2** methylation might act as a novel diagnosis standard for these cancer sites, thereby validating the efficacy and accuracy of our analysis methods.

### Candidate Methylation Patterns Discriminating the Origin Tissues of Tumor Cells

For the predicted features generated by the mRMR and MCFS algorithms, we apply two typical decision tree algorithms, namely, RIPPER and PART, to reveal the potentially associated methylation rules. For each rule group, we choose a few representative rules, as listed in Table 4, for detailed discussion as shown below.

**Table 4:** Representative rules for classifying tumor cells from different tissues.

**Index**	**Feature ranking method**	**Rule learning algorithm**	**Rule** **conditions**	**Classified tissues**	**Marker** **genes**
Rule 1	mRMR	RIPPER	(cg03977657 ≤ 0.099) and (cg01149192 ≤ 0.666) and (cg25593954 ≥ 0.757)	Aerodigestive tract	LAMB3, MGAT1, SPOP
Rule 2	mRMR	RIPPER	(cg00983904 ≥ 0.833) and (cg24393316 ≤ 0.090) and (cg04976330 ≥ 0.777)	Lung	IFFO1, FOXE1, PUM1
Rule 3	MCFS	RIPPER	(cg22609576 ≥ 0.084) and (cg00879790 ≤ 0.134)	Digestive system	TRIM15, SPG20
Rule 4	MCFS	RIPPER	(cg20783697 ≤ 0.300)	Blood	BZRAP1
Rule 5	mRMR	PART	(cg22203219 ≤ 0.460) and (cg16419724 > 0.408) and (cg08454824 > 0.683) and (cg13466284 > 0.577) and (cg16798247 ≤ 0.754) and (cg00989853 > 0.900)	Nervous system	IFFO1, MARVED2, ERICH1, SFN, ELMO1, IRF6
Rule 6	mRMR	PART	(cg20783697 ≤ 0.698) and (cg01951274 ≤ 0.130)	Blood	BZRAP1, MIR142
Rule 7	MCFS	PART	(cg02505827 ≤ 0.184) and (cg19519643 ≤ 0.785) and (cg00112091 > 0.118) and (cg05607401 ≤ 0.864)	Urogenital system	TEAD1, GMFG, MARVELD2
Rule 8	MCFS	PART	(cg02505827 ≤ 0.150) and (cg23229016 ≤ 0.645)	Skin	MARVELD2, RPS6KA1

Combining the mRMR and RIPPER algorithms, we obtain 47 associated rules, and ample recent reports can validate the accuracy and efficacy of these identified rules. For instance, the combination of three gene methylation status, namely, **LAMB3** (cg03977657) and **MGAT1** (cg01149192) hypomethylation, and **SPOP** (cg25593954) hypermethylation, is a specific feature of digestive tract and respiratory tract tumor (Rule 1 in Table 4). **LAMB3** is a component of laminin-5, an essential extracellular glycoprotein contributing to the most biological processes of basement membrane, including cell migration (Santamato et al., 2011), signal transduction (Filla et al., 2014), and tumorigenesis (Rani et al., 2013). Early in 2011, hypomethylation induced by abnormal overexpression of **LAMB3** contributes to gastric tumor procession (Kwon et al., 2011). **SPOP** methylation rate is correlated with colorectal tumor survival (Zhi et al., 2016). A study on colorectal tumor has validated that the upregulation of the hedgehog signaling pathway in colorectal tumor mediated by **SPOP** hypermethylation promotes tumor migration (Zhi et al., 2016). Another rule (Rule 2 in Table 4) for lung tumor classification also verifies the efficacy of our results. Three differentially methylated genes, **IFFO1**, **FOXE1**, and **PUM1**, were predicted as signatures for lung tumor. **IFFO1** methylation participates in non-small-cell lung cancer (Feng et al., 2017), and **PUM1** is an RNA-binding protein gene that participates in multiple biological processes, such as translational regulation (Lin et al., 2019) and cell development (Lin et al., 2018). Various recent studies have illustrated that **PUM1** functions in lung tumor. **PUM1** can inhibit the proliferation of non-small-cell lung cancer cells via targeted by MiR-411-5p (Xia et al., 2018) and can mediate the interaction between p27 and MiR-221, which leads to the deterioration of non-small-cell lung cancer (Fernandez et al., 2015). Therefore, the hypermethylation of **PUM1** is an important epigenetic characteristic for non-small-cell lung cancer diagnosis.

A total of 48 rules are obtained using the MCFS and RIPPER algorithms. Taking methylation rules for the classification of digestive system tumor as an example (Rule 3 in Table 4), differentially methylated genes **TRIM15** (cg00879790), and **SPG20** (cg22609576) are identified as signatures. **TRIM15** is an essential focal adhesion protein mainly distributed in the duodenum and the small intestine (Fagerberg et al., 2014). In general, such gene function acts as important regulatory component in biological processes, including focal adhesion turnover and cell migration (Uchil et al., 2014). **TRIM15** contributes to various types of digestive system tumor, including colon tumor (Lee et al., 2015) and gastric adenocarcinoma (Chen et al., 2018b). Moreover, specifically abnormal hypermethylation on **TRIM15** has been detected in the gastric cancer genome (Cheng et al., 2014), confirming the potential of **TRIM15** methylation as a candidate signature for gastric cancer diagnosis. Another candidate gene, **SPG20**, is a potential epigenetic signature for colorectal cancer (Rezvani et al., 2017). Hypermethylation-induced **SPG20** silencing directly contributes to the cytokinesis of colorectal cancer cells (Lind et al., 2011). This evidence validates the efficiency of this rule. In addition, according to another rule (Rule 4 in Table 4), gene **BZRAP1** was used to contribute to the identification of blood samples. Hypomethylation of such gene is positively correlated with the blood samples. **BZRAP1** has been identified in various blood cells especially in monocytes (Yasui et al., 2007; Jyonouchi et al., 2011). Therefore, the hypomethylation of such gene as a biomarker for blood tissues (blood cells) is quite reasonable.

Similarly, 72 rules are generated by the mRMR and PART algorithms. Substantial evidence supports the accuracy of these rules. For instance, we extract a rule (Rule 5 in Table 4) of methylation pattern for nervous system tumor, where **IFFO1** (cg22203219), **MARVED2** (cg16419724), **ERICH1** (cg08454824), **SFN** (cg13466284), **ELMO1** (cg16798247), and **IRF6** (cg00989853) were identified as candidate signatures. Among them, **SFN** and **ELMO1** have been widely reported to associate with nervous system tumor process. The hypermethylation of **SFN** is a reliable biomarker for neuroblastic tumor diagnosis (Banelli et al., 2005, 2010). **ELMO1** encodes a cell motility protein that contributes to glioma cell invasion. Recent research has also confirmed that **ELMO1** presents abnormal methylated status in glioblastoma (Michaelsen et al., 2018). Furthermore, a specific rule (Rule 6 in Table 4) for blood uses two effective parameters, **BZRAP1** (cg20783697) and **MIR142** (cg01951274). As for **BZRAP1**, the hypomethylation of such gene has been discussed above to be correlated with blood samples, validating such rule. As for microRNA142, it and microRNA-29a have been identified as potential biomarkers for myeloid differentiation and acute myeloid leukemia, which would be regarded as a potential biomarker for the identification of blood tissue.

Finally, for the combination use of MCFS and PART algorithms, 80 rules are generated by the MCFS and PART algorithms. These rules can be validated by recent publications. For instance, we use the dys-methylation status of **TEAD1** (cg00112091), **GMFG** (cg05607401), and **MARVELD2** (cg02505827) as the diagnostic signatures for urogenital system tumor (Rule 7 in Table 4). Methylation of the **MARVELD2** gene could be used to classify multiple different tumor types (Wang et al., 2009). With regard the relationship between **MARVELD2** and urogenital system tumor, this gene is highly expressed in the epididymal epithelium and contributes to its integrity (Mandon and Cyr, 2015). Hence, the mutation on **MARVELD2** may influence urogenital tumorigenesis. Meanwhile, **GMFG** is a member of the glia maturation factor family, and it has been validated to mediate angiogenesis by regulating the expression of STAT3 and VEGF (Zuo et al., 2013). Recent literature has confirmed that **GMFG** might contribute to the migration and invasion of ovarian cancer cells (Zuo et al., 2014). **TEAD1**, a ubiquitous transcriptional factor, acts as a transcriptional repressor in placental cells (Kessler et al., 2008). Hence, its increased level of methylation may lead to transcriptional alterations, further inducing tumorigenesis. Another specific rule (Rule 8 in Table 4), involving **MARVELD2** (cg02505827) and **RPS6KA1** (cg23229016), contributes to the identification of skin tissue. As discussed above, **MARVELD2** has been reported to contribute to the classification of multiple tumor subtypes at methylation level (Wang et al., 2009), including skin cancer (Jonckheere and Van Seuningen, 2018). As for **RPS6KA1**, the hypomethylation of such gene has also been reported to be functionally correlated with tumorigenesis in skin by interacting with gene RB1 (Mcevoy et al., 2014).

Limited by the page restrictions of this article, we are unable to discuss all results. Nevertheless, we have shown the efficiency of our computational methods for identifying novel tumor-specific epigenetic signatures. We widely validate the accuracy or relevance of our highly ranked methylation signatures and associated rules via literature studies. Our analysis method provides new insights into the precancerous diagnosis of different tumor types.

### Functional Enrichment Analysis for the Common Genes From mRMR and MCFS

**Figure 6:**
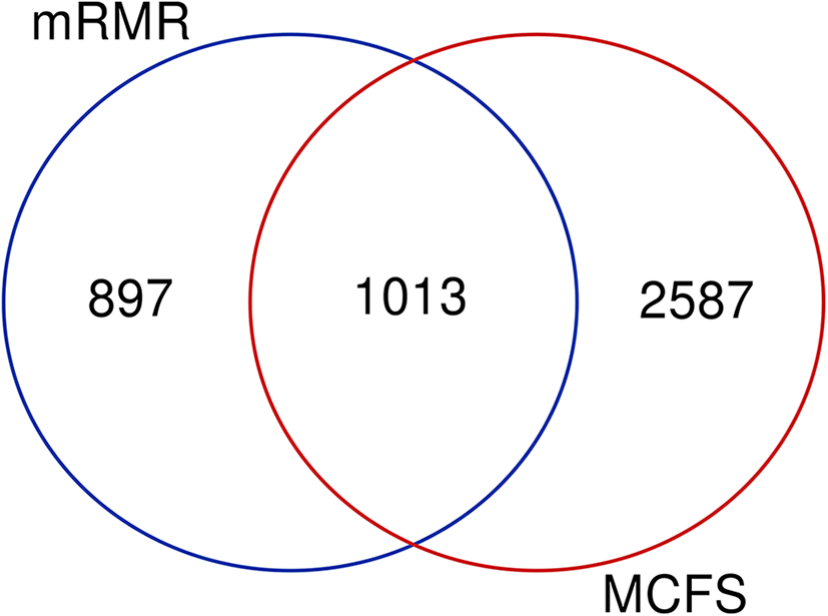
Venn diagram to show two marker gene sets yielded by mRMR and MCFS.

Based on the feature list yielded by mRMR, SVM with top 1,910 features provides the best performance, whereas SVM with top 3,600 features produces the best performance based on the list generated by MCFS. A Venn diagram is plotted in Figure 6 to show the difference of these two feature subsets. There are 1,013 common features (methylation sites), corresponding to 470 genes, which are provided in Table S9. For capturing more biological or pathogen understanding on these common marker genes, we carry on the functional enrichment analysis on GO and KEGG. Results are provided in Table S10.

On one hand, for gene ontology enrichment, GO: 0098609 (cell-cell adhesion), GO:0007155 (cell adhesion), and GO: 0022610 (biological adhesion) are the top GO (BP) terms for the enrichment pattern of common marker genes. According to recent publications, early in 1998, the inactivation of E-cadherin-mediated cell adhesion has been reported to participate in the progression of multiple cancer subtypes (Hirohashi, 1998). Further detailed studies confirm that cell-cell adhesion plays irreplaceable roles for the tumorigenesis, although the expression level and detailed contributions are actually not all the same in various cancer subtypes (Birchmeier et al., 1993), e.g., in primary and metastatic lung cancer (Böhm et al., 1994). Next, we identify various GO (CC) terms describing the cell-cell junction, such as GO: 0030054 (cell junction), GO: 0005911 (cell-cell junction), and membrane associated GO terms, including GO: 0044459 (plasma membrane part), GO: 0031226 (intrinsic component of plasma membrane), and GO: 0005887 (integral component of plasma membrane). As analyzed above, cell adhesion is a quite important biological processes for identification and discrimination on different cancer subtypes (Birchmeier et al., 1993). Considering that cell junction is functionally correlated with cell-cell adhesion (Kametani and Takeichi, 2007), it is also reasonable for marker genes to enrich in these related functions. Plasma membrane has also been reported to participate in multiple cancer subtypes (Leth-Larsen et al., 2010), especially in breast cancer (Razandi et al., 2000) and colon cancer (Kakugawa et al., 2002). Furthermore, for GO (MF) terms, GTPase function associated GO terms have been widely screened out, including GTPase regulator activity GO:0005096 (GTPase activator activity), GO:0017048 (Rho GTPase binding), and GO:0051020 (GTPase binding). GTPase function and its related biological processes have been identified in multiple cancer subtypes (Wang et al., 2003; Sethakorn and Dulin, 2013), and have been confirmed to play different regulatory roles for tumorigenesis in different cancer subtypes (Wang et al., 2003).

On the other hand, for KEGG pathways, the top KEGG pathways are just the same as the top biological processes describing the cell junction and adhesion hsa04520 (adhesions junction) and hsa04510 (Focal adhesion). There are other key pathways found, e.g., hsa04015 (Rap1 signaling pathway) and hsa04151 (PI3K-Akt signaling pathway). According to recent publications (Kooistra et al., 2007), Rap1 together with its regulatory pathways have been identified as a key regulator for cell-cell junction formation, so that, it is quite reasonable to regard Rap1 signaling pathway as a discriminative pathway for different cancer subtypes. As for PI3K-Akt signaling pathway, it is actually one of the most famous tumor associated pathways, which has been identified to be pathogenic in multiple tumor subtypes, including breast cancer (Berns et al., 2007), B-cell lymphoma (Lannutti et al., 2011) and endocrine tumor (Robbins and Hague, 2016). Many studies confirm that actually in different tumor subtypes, the activation status and drive contribution of such pathway on tumorigenesis may be not always the same (Boyault et al., 2012).

## Conclusions

This study investigates the methylation data of tumor cell lines from 13 tissues. Several machine leaning algorithms are employed to provide deep insights into the data. Some methylation-associated genes and their dys-methylated patterns are extracted. The genes may be novel biomarkers for discriminating different tumor cell lines and the patterns can provide a clear picture on the methylation levels of tumor cell lines in different tissues. The findings reported in this study may be novel materials for the study of tumor cell lines.

## Data Availability Statement

The datasets for this study can be found in the Gene Expression Omnibus [https://www.ncbi.nlm.nih.gov/geo/query/acc.cgi?acc=GSE68379].

## Author Contributions

TH and Y-DC designed the study. SZ, TZ, BH, and LC performed the experiments. SZ, Y-HZ, KF, ZN, and JL analyzed the results. SZ, TZ, and BH wrote the manuscript. All authors contributed to the research and reviewed the manuscript.

## Conflict of Interest

The authors declare that the research was conducted in the absence of any commercial or financial relationships that could be construed as a potential conflict of interest.

## Supplementary Material

Table S1 Ranked features by mRMR.

Table S2 Ranked features by MCFS.

Table S3 Performance of IFS with SVM, PART, and RIPPER when using different numbers of features ranked by mRMR.

Table S4 Learned classification rules by PART based on the ranked features by mRMR.

Table S5 Learned classification rules by RIPPER based on the ranked features by mRMR.

Table S6 Performance of IFS with SVM, PART, and RIPPER when using different numbers of features ranked by MCFS.

Table S7 Learned classification rules by PART based on the ranked features by MCFS.

Table S8 Learned classification rules by RIPPER based on the ranked features by MCFS.

Table S9 Common features (methylation sites) and their corresponding genes detected by mRMR and MCFS.

Table S10 Functional enrichment analysis of common genes detected by mRMR and MCFS.
